# Persisting Prejudice: Measuring Attitudes and Outcomes by Caste and Gender in India

**DOI:** 10.26812/caste.v1i2.172

**Published:** 2020-10

**Authors:** Amit Thorat, Nazar Khalid, Nikhil Srivastav, Payal Hathi, Dean Spears, Diane Coffey

**Affiliations:** 1 Centre for the Study of Regional Development, Jawaharlal Nehru University, Delhi; 2 Department of Demography, University of Pennsylvania, Philadelphia; 3 LBJ School of Public Affairs, University of Texas, Austin, Austin; 4 Departments of Demography & Sociology, University of California, Berkeley; 5 Population Research Center & Department of Economics, University of Texas, Austin; 6 Population Research Center & Department of Sociology, University of Texas, Austin

**Keywords:** Gender, domestic violence, inter-caste marriage, religion, attitudes, India

## Abstract

Nearly seventy years after India adopted one of the most progressive constitutions in the world ensuring equality for all its citizens irrespective of caste, class, race, and gender, the mind-set of its vast majority Indian remains steeped in gender and caste bias. Results from a new telephonic survey confirm persistence of conservative gender and caste attitudes in Indian society. High proportions of men and women across all social groups disapprove of women working outside their homes, consider it ’acceptable for husbands to beat their wives’, and would object to relatives marrying a Dalit person. Analyzing data from the National Family Health Survey and the India Human Development Survey, it has been found that outcomes associated with these attitudes are even more conservative: a smaller fraction of women work than those who feel it is okay to step out of the house for work; a larger fraction of women experience violence in marriage than men who consider marital violence acceptable, and an even smaller fraction of people have inter-caste marriages than people who say they would not oppose such an alliance. An overwhelming majority is opposed to an inter-caste marriage with a Dalit in the family. With a few exceptions, the attitudes and outcomes we studied vary, surprisingly, little by respondent gender, caste, and religion. Dr.Ambedkar’s legacy is indeed unfinished–people from all backgrounds must continue to work for the equality and dignity of women and Dalits.

## Introduction

In his 1951 resignation speech, Dr. B.R. Ambedkar explains that he was leaving the Cabinet because neither the Constituent Assembly nor the Prime Minister would support his draft of the Hindu Code Bill. Dr. Ambedkar’s draft of the Bill would have abolished the caste system, provided legal recognition for inter-caste marriages, and allowed women to divorce their husbands and inherit property. In the speech, he states:

“To leave inequality between class and class, between sex and sex, which is the soul of Hindu Society untouched and to go on passing legislation relating to economic problems is to make a farce of our Constitution and to build a palace on a dung heap. This is the significance I attached to the Hindu Code.”

Unfortunately, the ‘dung heap’ to which Dr. Ambedkar refers still exists today. Despite his struggle for equality and dignity of Dalits and women, caste and gender discrimination are widespread. Exactly how deep is the heap? There are many ways of answering this question. One way is by studying the lived experience of Dalits and women. Personal accounts help us understand what discrimination and prejudice mean to people who experience it.^1^

Another way is by quantitatively describing the outcomes of discriminatory social processes: how many women are part of the paid labor force? How many experience violence in their marriages? How many marriages are inter-caste? A third way of understanding discrimination is to ask people to report their attitudes towards marginalized groups. In this paper, we present novel results from a social attitudes phone survey and analyze whether attitudes about women and Dalits differ by gender, caste, and religion. This way of measuring discrimination is less common, but makes valuable contributions to our understanding of the discriminatory processes.

Some social scientists may argue that when outcomes are measured attitudinal data has little to add to studies of discrimination. However, we see several benefits of using attitudinal data to complement analysis of outcomes. First, it allows us to learn directly from those who discriminate or perpetrate violence. For example, men who beat their wives might be more likely to say that they think that abuse is acceptable even if they will not admit to doing it themselves. Further, attitudinal data allows us to assess how much support or opposition those who try to change the status quo might face. For example, if social approval for marrying outside one’s caste is low, from within and outside one’s family (Rajadesingan et al, 2019), inter-caste couples may face difficulties getting legal approval for their weddings, or finding a place to live. Moreover, outcomes will continue to be adverse so long as attitudes persist. Once we understand the nature and extent of people perceptions, we can then try and think of interventions and policy tools to change or mitigate social perceptions and mindsets. This paper focuses on three attitude-outcome pairs of indicators:

1. What proportion of women work for pay and whether people think women should work for pay;
2. What proportion of women experience physical violence at the hands of their husbands and whether people think it is acceptable for a husband to beat his wife; and
3. What proportion of people have inter-caste marriages and whether people say they would object if their relative were to marry someone from a Dalit caste.

For each of these pairs, responses to the attitude question cannot be seen as a direct comment on the outcomes that we study. They however reflect the prevalent mindscape of social attitudes. The questions, which are discussed below, ask about something slightly different than the outcome. Despite these differences, analyzing these attitude-outcome pairs presents the opportunity to reflect on how attitudes towards women and Dalits remain extremely conservative, and how outcomes often change in spite of stated attitudes.

Many India observers have expected changes in social and inter-personal attitudes to follow from the country’s path of economic development. Yet, as Dr. Ambedkar had observed almost seventy years ago, economic policies alone will not bring equality and dignity for women and Dalits. Although India has been on the path of economic growth--opening up its closed economy and transforming it into a liberal global one-- it is clear that much work remains to be done to end patriarchy and caste discrimination. Dr. Ambedkar’s legacy is yet unfinished.

## Data, Measures, and Methods

### Categorization of Social Groups

With some modification, we follow the India Human Development Survey’s (IHDS) categorization of social groups (Desai et al., 2012) for within-India comparisons. The IHDS categorizes Indian population as Brahmin, Forward caste, Other Backward Class (OBC), Dalit (Scheduled Caste or SC), *Adivasi* (Scheduled Tribe or ST), Muslim, Christian, or other. In the IHDS, Brahmins, Forward Castes, and OBCs are largely Hindu. Muslims and Christians of any caste background are considered Muslim or Christian. We note that IHDS did collect caste categories for Muslims and Christians, but suggests this grouping for some analyses.

The social attitudes data we use is from a mobile phone survey called Social Attitudes Research, India (SARI). Because SARI’s sample sizes are not large enough to analyze people from religious groups other than Hindus and Muslims, we have modified the IHDS categorizations as follows. This, in both the NFHS and SARI data, we look at:

- Scheduled Castes (only Hindus)
- Scheduled Tribes (Hindu or Muslim)
- Other Backward Classes (Hindu)
- General caste (Hindu)
- Muslim (all castes)

We note that for international comparisons, we use data on all of India, including Christians, Sikhs, and people of other religions. People belonging to religions other than Hinduism and Islam make up only about five percent of the Indian population.

### The Social Attitudes Research, India (SARI) Survey

SARI is a mobile phone survey designed by the authors to collect data from adults, ages 18 to 65. Data were collected in Delhi, Uttar Pradesh, Rajasthan, Mumbai, Maharashtra, Bihar, and Jharkhand between 2016 and 2018. Respondents in each of these samples were interviewed by a person of the same sex. During the period in which interviews were being conducted in Mumbai, there were no Marathi speaking interviewers on the SARI team. Therefore, no women were interviewed in Mumbai. For this reason, the Mumbai sample has not been used for our study.

SARI uses random digit dialing, a method that is common for recruitment of representative phone survey samples. In each place-specific mobile circle in India, the Department of Telecommunications issues a certain number of five-digit ‘series’ to each phone company to use as the first five digits of the phone numbers they provide to consumers. SARI generates potentially active phone numbers for interviewers by concatenating the five-digit series (listed in proportion to the number of subscribers to that phone company) with five randomly generated digits to form 10-digit mobile phone numbers. Surveyors call these numbers in a random order. Approximately half of the phone numbers generated in this way are active, as opposed to not in use, switched off, or unreachable.

At the beginning of the call, the interviewer asks the person who answers the phone to list all of the men or women in the household (depending on the sex of the respondent who is supposed to be interviewed). Survey respondents are selected randomly from the household listing by Qualtrics software. Within-household respondent selection ensures that even individuals who do not own their own mobile phones have a chance to be interviewed. In Delhi, Uttar Pradesh, Rajasthan, Bihar, and Jharkhand, most respondents take the survey in Hindi. However, some respondents choose to take the survey in local languages. Surveyors who speak Marwari, Bagdi, Maithili, and Bhojpuri interviewed respondents who did not speak Hindi. Women were more likely to be interviewed in local languages than men. In Maharashtra, most respondents answered in Marathi, but some chose to take the survey in Hindi.

Since individuals from some demographic groups are more likely to respond to the survey than others, we weight our results using statistical weights created from the population data for the state of Rajasthan provided in the 2011 India Census. Weights account for the intersection of sex, place (i.e. urban/rural), education, and age.

Table 1 gives details about the period when the SARI survey was done in each place, and the sample sizes of men and women from the social groups studied in this paper.^2^ Because this paper is interested in differences by social group, we restrict the data to the afore-mentioned groups. Sample sizes for other social groups are too small to draw reliable conclusions in the SARI data. Response rates for the SARI survey range from about 20-25 percent across the six places we study. These response rates are of course much lower than for face to face surveys in India. To put the response rates in context, a study of the Pew Research Center’s 2012 phone surveys in the US found an average response rate of nine percent (Kohut et al., 2012). Thus, response rates for SARI are quite high. As long as (conditional on sex, place, education, and age) those who answer the survey do not have different views than those who do not answer, the sample will accurately represent the population.

Table 1 also shows the percentage of households in NFHS that had mobile phones in 2015. This information is included to show that although some households were not included in the sampling frame because they lacked a mobile phone, the vast majority were. Further, the NFHS was conducted over a two-year period, allowing us to observe that households interviewed later in time within the same states were more likely to own a mobile phone. For instance, in Bihar, where data were collected over six months in 2015, households interviewed in the last month were nearly four percentage points more likely to have a mobile phone than households interviewed in the first month. This suggests that coverage would have continued to increase after 2015, and would have been substantially higher when SARI data were collected.

Further information about survey design and data collection, as well as the strategies we use to reduce non-sampling error, can be found in Coffey et al. (2018) and in the online survey documentation (r.i.c.e., 2017).

### The Demographic & Health Surveys and the National Family Health Survey 2015

The Demographic and Health Surveys (DHS) are an international collaboration between USAID and governments and research institutions in low and middle income countries. Since the 1970s, the DHS have measured health, fertility, mortality, and gender empowerment. Because many low and middle income countries lack vital registration systems, the DHS provide the most reliable measures of fertility and mortality for some countries. The DHS data are publicly available at www.dhsprogram.com.

**Fig. 1:**
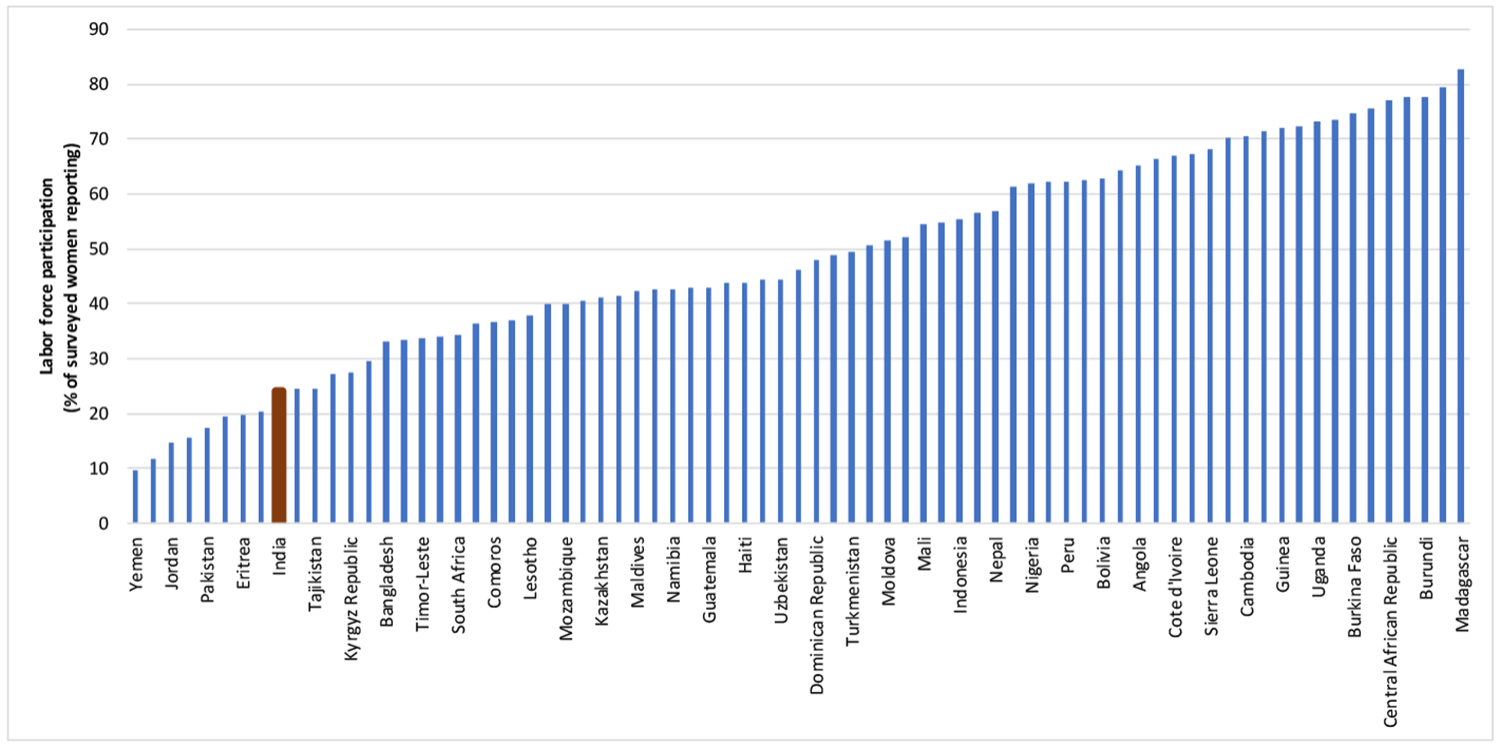
Women’s Labour Force Participation in India Compared with other Countries *Data Source:* Demographic and Health Surveys Statcompiler.

The international comparisons in this paper use the most recent DHS for each country shown in Figures 1 and 3. Because the DHS uses many of the same questions in every country, the results are comparable across countries. We used the DHS Statcompiler (https://www.statcompiler.com/) to compute the fraction of women in the labour force and who experienced marital violence in each country. The same sample restrictions apply to each country. For the fraction of women who are working in the last twelve months, all women (ages 15-49) are included regardless of whether or not they are married. For the fraction that experienced marital violence in the last 12 months, only married women (ages 15-49) are included.

India’s DHS is called the National Family Health Survey (NFHS). The NFHS was last collected in 2015 (IIPS & IFC, 2017) and is representative at the district level for many variables. It is a multi-stage, clustered survey that collects a number of variables related to health and nutrition, including height, weight, communicable and non-communicable disease, and HIV prevalence. It also collects a number of variables relating to women’s status, including their education, work, economic situations, decision-making power, and experiences of physical, sexual, and emotional violence. The NFHS 2015 collected data on work for 122,351 women (ages 15-49), and data on experiences of physical violence for 66,013 women in the same age group who were married at the time of the survey. This includes women from all social groups.

### The India Human Development Survey, 2011

The India Human Development Survey (IHDS) is a nationally representative, clustered, multi-stage panel survey of over 42,000 households which was conducted in 2005 and 2012 (Desai et al., 2012). It is a joint undertaking of the National Council for Applied Economic Research (NCAER) and the University of Maryland. This is the only panel in India that collects data on household incomes and consumption expenditure amongst data on many health and social welfare indicators. It has a number of novel measures of women’s status and caste prejudice. The data are publicly available at https://ihds.umd.edu/data-download. This paper uses data on married women from Hindu and Muslim backgrounds. There are 39,523 such women in the data.

### Measurement of Gender and Caste Outcomes and Attitudes

We analyze three measures of outcomes, and three corresponding measures of attitudes. The outcomes are measured by the NFHS and IHDS and the attitudes are measured by the SARI survey. Table 2 enlists each question along with sample description and the response options. Although it is not noted in Table 2, the aforementioned sample restrictions with respect to categorization of social groups apply i.e., for within-India analyses, we drop individuals who do not fit into anyone of the social groups described above.

## Methods

Figures showing comparisons of outcomes in India to other countries present weighted proportions. Figures showing intra-India comparisons present ninety-five percent confidence intervals for proportions in order to assess whether outcomes and attitudes are statistically significant by subgroup. For both of NFHS and IHDS, we rely on the asymptotic normality of the large sample to compute clustered standard errors using an identity link function. For SARI, which has a smaller sample and is not clustered but instead is interpreted as a weighted simple sample, we compute standard errors using a logit link function. All results use population weights. For the SARI analyses, we construct population weights for the region of states for which a particular question was asked.

## Results

### Women’s Work and Movement Outside Home

Figure 1 shows the proportion of women in India who worked in the 12 months preceding the survey as compared to their counterparts in other countries according to recent DHS. Although different ways of estimating women’s labour force participation will yield slightly different results, the overall take-away is similar to that of the papers we reviewed in the Introduction section: In India, women labour force participation is low as compared to other countries. Only about a quarter of women worked in the year before the survey. The fact that three quarters of women or seventy five percent did not work suggests large economic losses, as well as a general environment of restrictions on women’s mobility and financial freedom. The work participation rate for women, as it stands now is part of a declining trend. It was thirty five percent in 2011 and now stands at around twenty three, placing India at the 12^th^ position from the bottom in world rankings.

### Does the Proportion of Women Who Work Vary by Social Group?

**Fig. 2:**
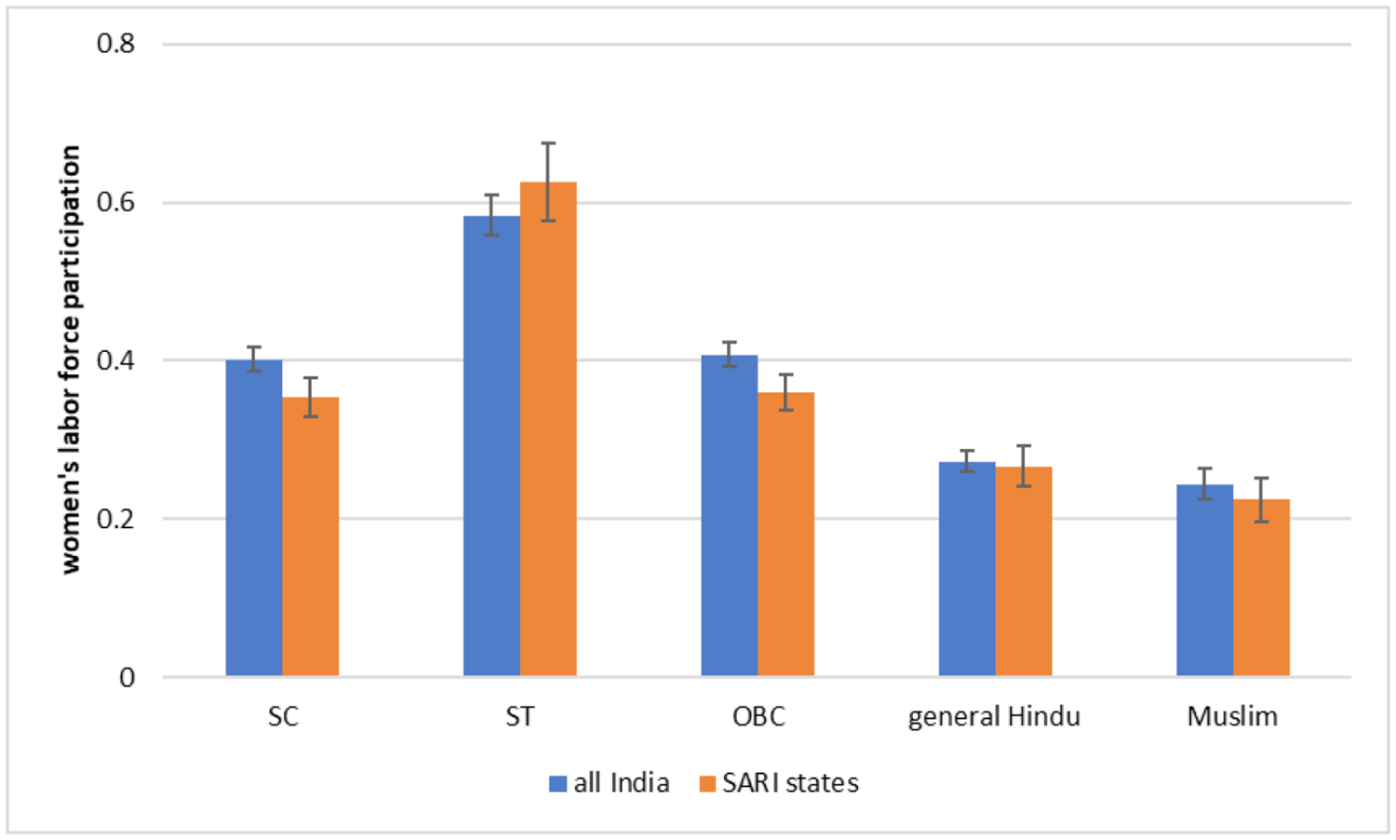
Women’s Labour Force Participation by Indian Social Groups *Data Source*: National Family Health Survey, 2015. SARI states are Delhi, UP, Bihar, Jharkhand, Maharashtra, and Rajasthan.

The presence of women in the labour force across social groups is depicted in Figure 2. On the whole the rates of participation are higher amongst Hindu women as compared to Muslim women. If we look at the differences in a regression framework, the difference in the proportion of women who work is greater when we account for the fact that Muslims are more likely to live in urban areas. Amongst Hindus, they are higher for OBCs, SCs, and STs than general caste women as well as Muslim women. After controlling for urban residence, we find that OBC Hindu women do not have statistically significantly different labour force participation than their general caste Hindu sisters, but the differences between SC and ST women and general caste Hindu women are substantial. Still, the raw rates of around forty percent for OBCs and SCs are lower than that of Nepal, where more than half of women work. Participation rates for ST women too are substantially higher, at approximately sixty percent. This is consistent with the prior literature discussed in the Introduction section. We note that levels of women’s work in the SARI states are similar to that in India as a whole. Women from socially and economically marginalized communities such as the SC (Dalits), ST (*Adivasis*) have had little choice but to work for wages, to supplement household incomes. This disadvantage is then visible, despite the observed secular fall in women’s work participation rates (Afridi et al., 2016), in the context of rising unemployment and slowing down of new jobs being generated, in falling but still higher work participation rates of ST and SC women.

### How do Attitudes Towards Women’s Work Vary by Social Group?

**Fig. 3:**
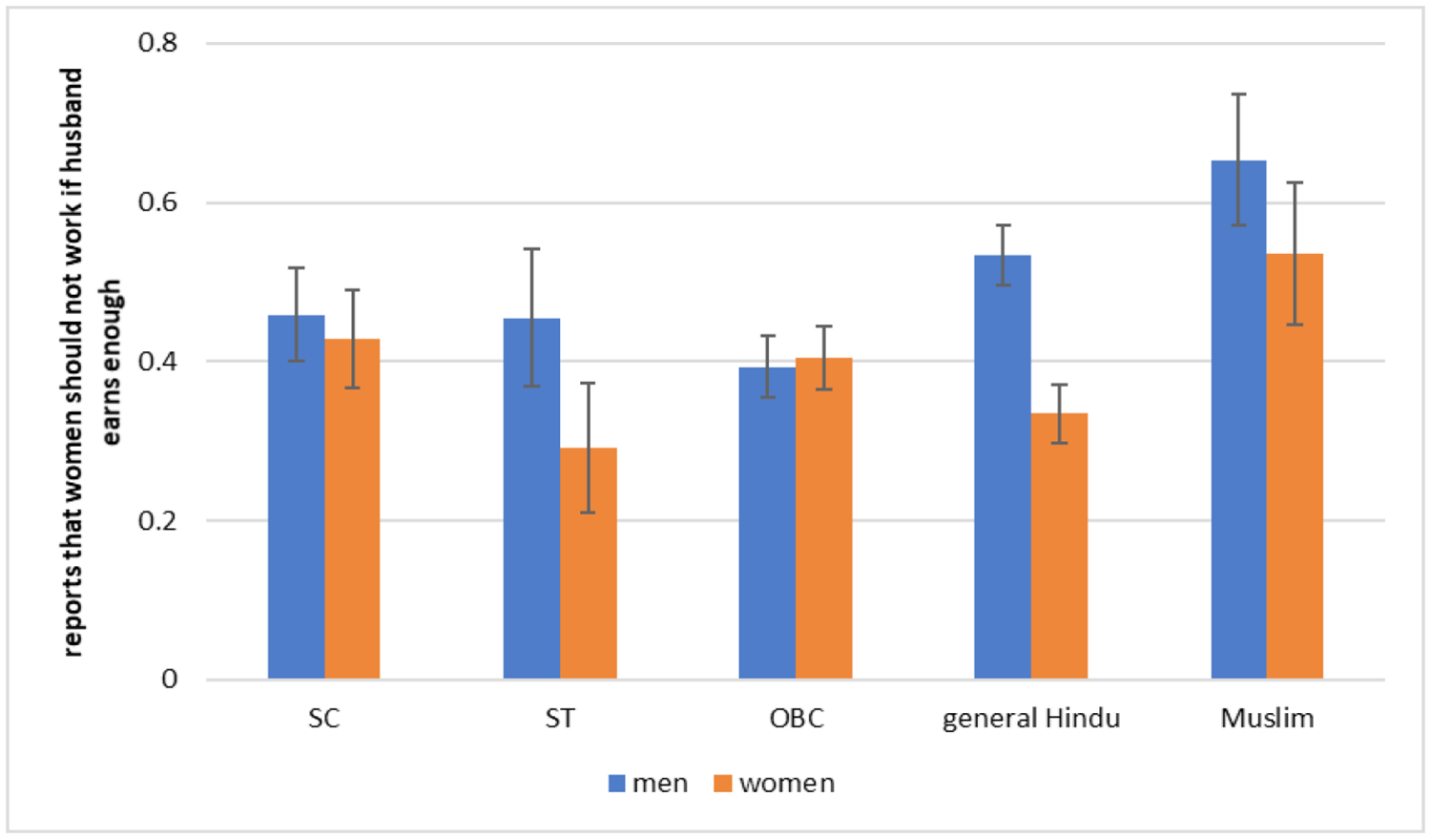
Attitude Towards Women’s Labor Force Participation by Indian Social Group *Data Source:* SARI (Delhi, UP, Bihar, Jharkhand, Maharashtra, and Rajasthan)

We note that many women from marginalized backgrounds work outside their homes due to economic necessity. However, the attitude question asked in the SARI survey refers to women whose husbands earn well. Figure 3 suggests that overall about half of adults say that women whose husbands earn well should not work outside home. There are, however, some statistically detectable differences by gender and social group. Except for OBC, for all groups, men in general have higher level of opposition to women’s work outside home. Muslim men and women and general caste men have the highest levels of opposition to women working outside home. SC men and women, OBC men and women, and ST men have statistically similar levels of opposition, which are lower than Muslims and general caste men, but higher than general caste women and ST women. The only groups for which there is a gender gap in opposition to women’s work outside are in the general castes and STs. When examined side by side Figure 2 and Figure 3 look almost like mirror images of each other, indicating groups where there is higher opposition to women working outside in case their husbands earn enough are also the ones that show lower level of female work participation rates. There is also some regional variation in attitudes towards women’s work: people in Maharashtra are less likely to disapprove of women working outside their homes than people in Jharkhand and Bihar.

### Experience of Marital Violence

**Fig. 4:**
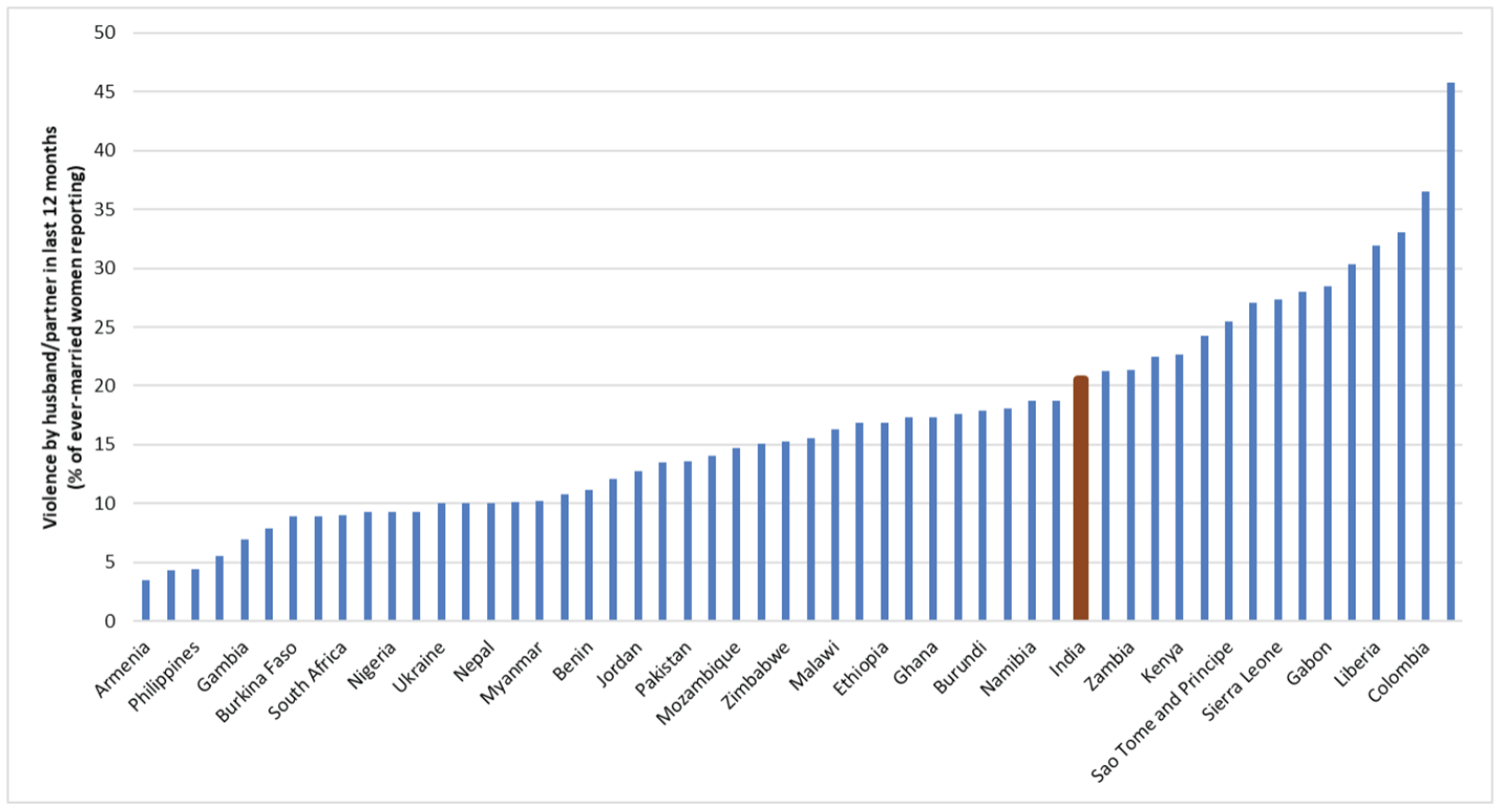
Physical Violence Against Married Women in India Compared to Other Countries *Data Source:* Demographic and Health Surveys Statcompiler.

More than one in five women in India report experiencing physical violence at the hands of their husbands in the twelve months before the survey (Figure 4). Although India is not the country wherein violence against women in marriage is most prevalent, it is more prevalent in India than in more than half of the countries taken up in the study. Often violence is normalized and internalized culturally as well as at an individual level and is more often than not an indication of deep-rooted intra-household, if not community and/or social patriarchal relationships.

**Fig. 5:**
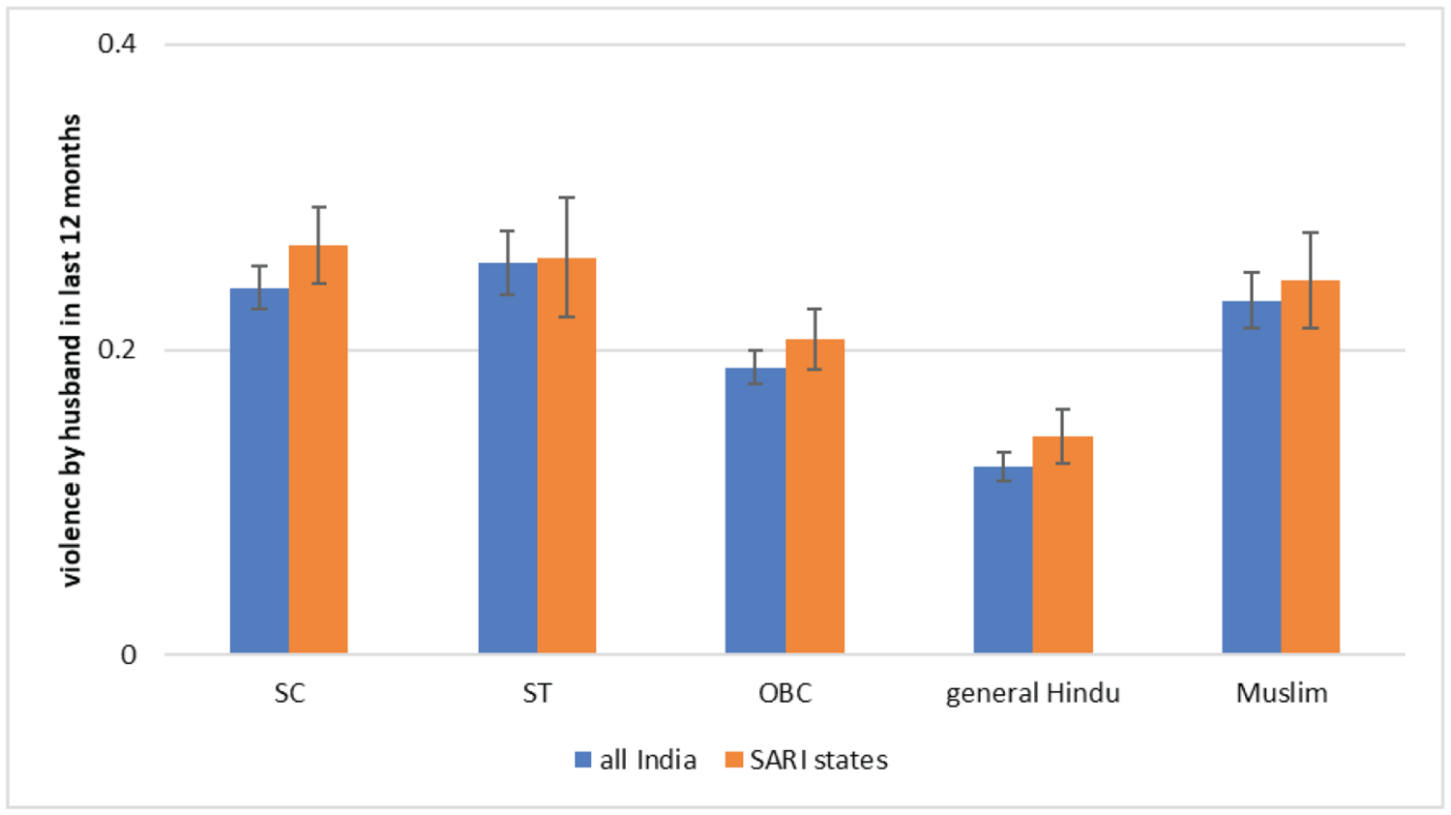
Women’s Experience of Physical Violence in Marriage by Indian Social Groups *Data Source*: National Family Health Survey, 2015. SARI states are Bihar, Jharkhand, and Maharashtra.

Are there differences in reported violence cross social groups? Figure 5 shows that with the exception of general caste women, levels of violence are similar for all other groups. Compared to other groups, general caste women are between five and ten percentage points less likely to report experiencing violence. Also, levels of physical violence in marriage are similar in the SARI states of Bihar, Jharkhand, and Maharashtra and in all of India.

**Fig. 6:**
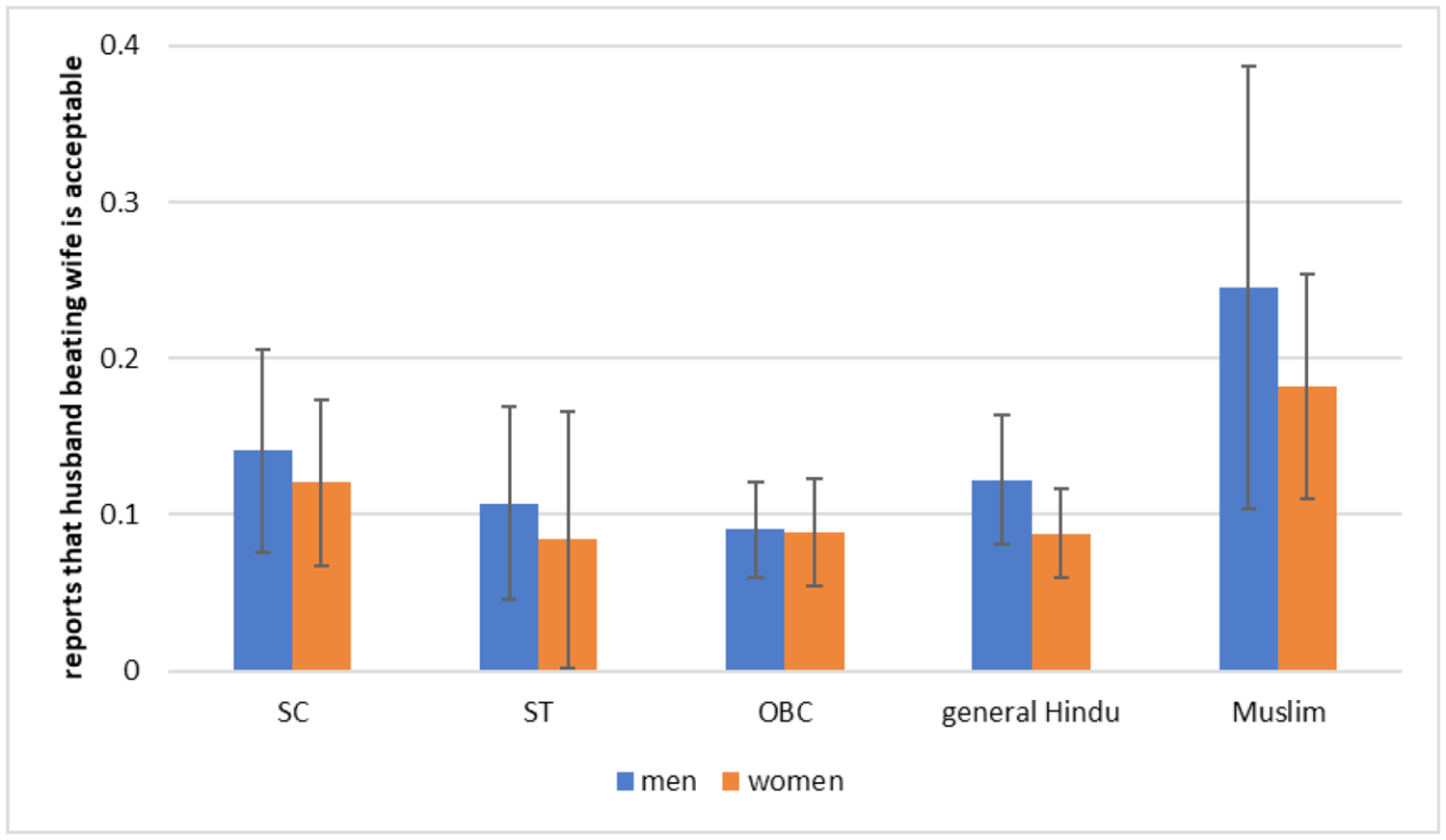
Attitudes Towards Marital Violence Suffered by Women Across Indian Social Groups *Data Source:* SARI (Bihar, Jharkhand, and Maharashtra)

Figure 6 shows the proportion of men and women in Bihar, Jharkhand, and Maharashtra who say that it is acceptable for a husband to beat his wife. Although data for all three states is clubbed, it is observed that the proportion of people who say it is acceptable is significantly higher in Bihar than in Jharkhand or Maharashtra. In each state, between ten and fifteen percent of both men and women say that it is acceptable. Surprisingly, there are neither any statistically significant differences in the percentage of men and women who consider marital violence acceptable, nor are there statistically significant differences across social groups. It is also notable that a smaller proportion of men say that it is acceptable for a husband to beat his wife than married women who say that they have experienced violence in marriage. This indicates that it is likely that lesser number of men and women acknowledge violence being acceptable than actually the case might be. At the same time lesser number of women accept facing violence by husbands, than actually might be the case.

### Inter-Caste Marriages

**Fig. 7:**
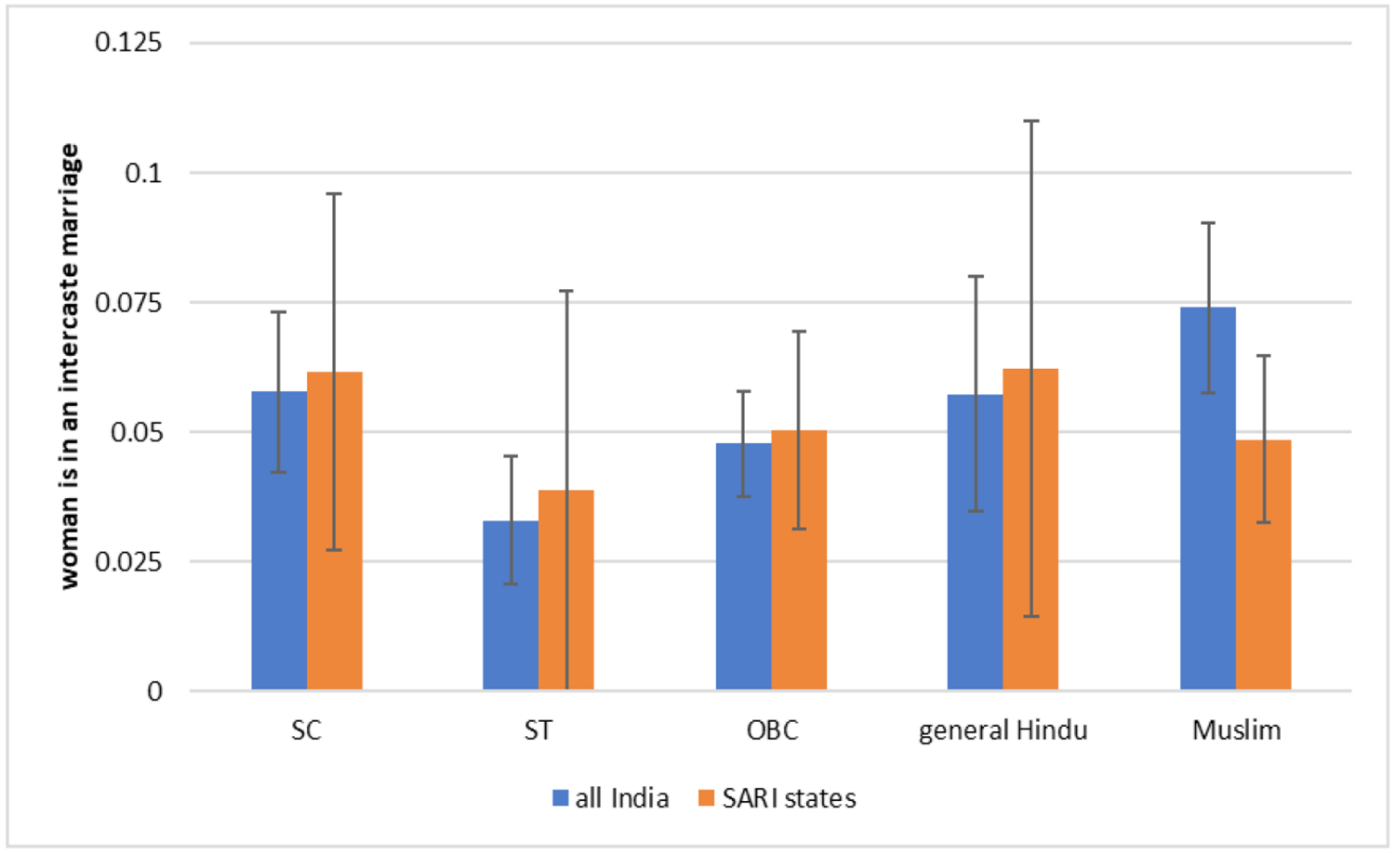
Proportion of Women Who Have had an Inter-Caste Marriage *Data Source:* India Human Development Survey. SARI states are Delhi, Uttar Pradesh, Rajasthan, Maharashtra, Bihar, and Jharkhand.

Consistent with the prior literature cited in the introductory section, Figure 7 uses IHDS data to show that only about five per cent of married women in our study are in inter-caste marriages i.e. their husbands’ families do not belong to the same caste as that of their parents. There is little variation across marital families of different social groups. We note that women who report inter-caste marriages may or may not belong to the broad category that they are grouped into here. Inter-caste marriages could be marriages across, say ST and General, or they could be marriages across sub-*jatis* (sub-castes or sub-groups) within a broad caste category. They are more likely to be within the same broad group but across different sub-groups.

The number of women in inter-caste marriages is quite low considering that the woman herself would have defined what it meant for her own natal family to be of a different caste than her husband’s family. It is also likely that most of the inter-caste marriages mentioned here are not marriages between Dalits and non-Dalits. As we discuss below, future studies could expand the existing literature by asking which caste or sub-caste the woman’s natal family belonged to.

**Fig. 8:**
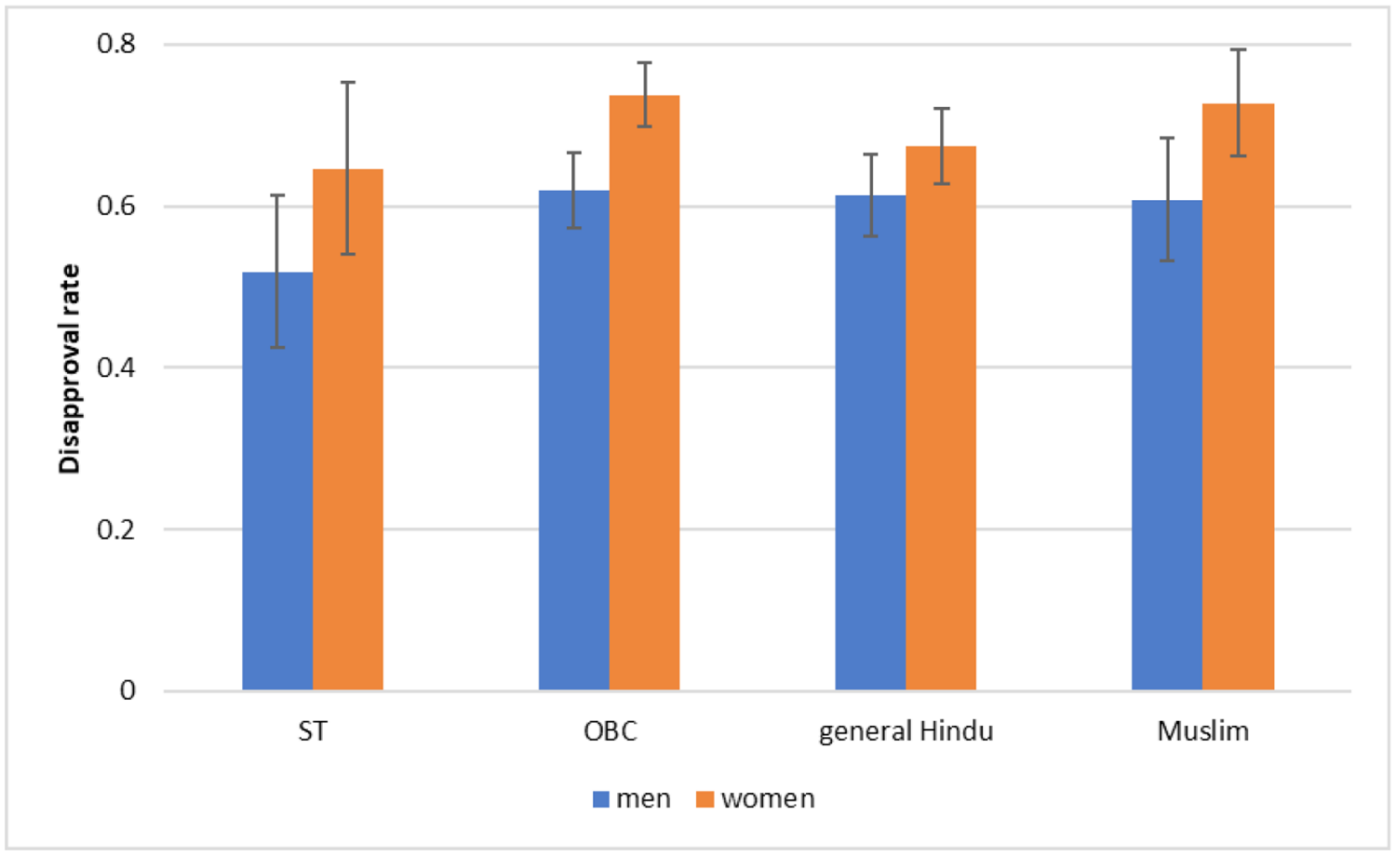
Attitudes towards Inter-Caste Marriage: Proportion Who Disapprove of a Relative Marrying a Dalit *Data Source:* SARI (Delhi, Uttar Pradesh, Rajasthan, Maharashtra, Bihar, Jharkhand

Figure 8 shows the proportion of men and women in each social group who report that they would object if a relative wanted to marry a Dalit person. We do not include Dalits in this analysis because they were not asked this question. There are not large differences across social groups in the proportion who say that they would object to a relative marrying a Dalit. In every social group, women are more likely to report that they would object than men, but the differences are not statistically significant when the data are broken up by social groups. Statistical significance in part depends on sample size; when the data are not broken up by social group (so the sample size is larger), women respondents are statistically significantly more likely to say they would object if a relative wanted to marry a Dalit person. This may be because they are actually more caste conservative, or it may be because they are less aware that saying that they would object could be considered a socially undesirable answer. Among women, the proportion is approximately seventy percent while it is about sixty percent among men.

This level of opposition to inter-caste marriage is quite disturbing as these perceptions are seen to be common across caste and religious identities. This lead us to the question whether this is internalization of the idea of purity and pollution across religious and caste norms and beliefs, or is it akin to a form of socially desirable normative behaviour?

## Discussion and Future Research

Other studies have observed the gender and caste outcomes for which we present results. For instance, Ray et al., 2017 note the low proportion of inter-caste marriages and find that inter-caste marriage is more likely to occur if the mother-in-law of the bride is more educated. Several recent studies have documented India’s low and declining female labour force participation (Chatterjee et al., 2015; Afridi et al., 2016; Klasen, 2017). Kishor & Gupta (2004), among others, have noted the high rates of violence that women in India face in their marriages.

What is novel about this study is the pairing of outcomes with social attitudes measured in the telephonic survey. The first thing that emerges from the new data is that stated attitudes towards women and Dalits are still quite conservative and do not seem to be abating at the rate we would hope for. The mismatch between attitudes and outcomes is not very large. However, stated attitudes are not as conservative as outcomes imply.

It is difficult to know exactly how to interpret the mismatch between outcomes and attitudes. Why do fewer women work than men, and why do fewer women than men say it is acceptable for a woman to work? One reason might be that even if a person has privately more liberal views, it is difficult to act on them until they are more widely shared due to social and economic costs on acting upon them. If women who consider working outside their homes, or people who are considering inter-caste marriages face social policing by conservative people in their networks, and they don’t have much support by way of economic and personal state protection, then they may not act on their more liberal attitudes.

Social desirability bias is almost certainly part of the explanation for the mismatch between attitudes and outcomes. Especially for attitudes around violence, it makes sense that some people who engage in violence would not be willing to admit it to an interviewer. Other research from the SARI data has discussed social desirability in reported attitudes and pointed to avenues for further research (Hathi et al., 2020a; Hathi et al., 2020b).

Another difficulty in interpreting a comparison of the outcome survey and the attitude survey is that they were done at different points in time. However, this is perhaps less of a concern in this context than others because there is evidence from other research that rates of women’s labour force participation and that of inter-caste marriages are changing slowly. There is less research on trends in marital violence against women. We hope that the fact that there is less approval for husbands beating their wives in the SARI survey than actual reports of it in the NFHS means that such behavior has decreased in the intervening 3-4 years. However, we think it is not plausible that a three to four years time difference could explain the entire gap.

A few directions for future research emerge from this study. First, it would be useful to better understand how the phone survey medium influences results. Would it make a difference if attitudes were measured in face to face surveys?

Further data analysis could be done to better understand why rates of labour force participation are low for women. Why do many general caste women say they support women working outside the home even though it is the group with the lowest labour force participation rate among women?

Is the fact that general caste women report experiencing less violence than women in other social groups a true difference, or due to differential reporting? The IHDS and NFHS have a series of questions around the excuses that men might make for violence against women that would provide deeper understanding of violence in marriage.

Finally, the inter-caste marriage rate in the IHDS was subjectively defined by the married woman. It would provide a clearer picture of discrimination against potential Dalit partners to know how many marriages specifically between Dalits and non Dalits occur.

Despite the need for further research on all of the indicators we have included in this study, it is clear that prejudice against women and Dalits remains persistently high, and that policies are needed to support those who digress from conservative social norms, either by choosing to work, choosing who they marry, or by leaving abusive partners.

## Figures and Tables

**Table 1:** Details of SARI data collection

Sample Sizes					
State	Year	Men	Women	Total	% HH Level Mobile Coverage in NFHS-2015
Delhi	2016	685	593	1278	98
UP	2016	746	752	1498	92
Rajasthan	2017	1570	1705	3275	94
Bihar	2018	1442	1980	3422	90
Jharkhand	2018	434	521	955	83
Maharashtra	2018	906	687	1593	90
total				12021	

**Table 2:** Questions on Gender Outcomes and Attitudes

Question	Sample Description for Within-India Analyses	Response Options
Outcomes (NFHS & IHDS)		
(NFHS) Have you done any work in the last 12 months?	women ages 15-49 in all states	Yes No
(NFHS) [In the last 12 months,] did your husband ever: push you, shake you, or throw something at you? twist your arm or pull your hair? slap you? punch you with his fist or with something that could hurt you? kick you, drag you, or beat you up? try to choke you or burn you on purpose? threaten or, attack you with a knife, gun, or any other weapon?	married women ages 15-49 in all states	Yes No
(IHDS) Is your husband’s family the same caste as your natal family?	married women ages 15-49 in all states	Yes No
Attitudes (SARI)		
In your opinion, should a married woman, whose husband earns a good living, work outside the home or not?	Adults ages 18-65 in Delhi, UP, Bihar, Jharkhand, Maharashtra, Rajasthan	Yes, she should work No, she should not work
Do you think it is right for a husband to beat his wife or not?	Adults ages 18-65 in Bihar, Jharkhand, Maharashtra	Yes No
If a close relative or someone in your family married someone from a Dalit caste would you oppose it or not?	Adults ages 18-65 in Delhi, UP, Bihar, Jharkhand, Maharashtra, Rajasthan	Yes, I would oppose it/ No, I would not oppose it

## Footnotes

1 See Valmiki (2003); Faustina (2014)

2 Sample sizes in this paper differ from sample sizes given in other papers that use SARI data because for these analyses we drop individuals who do not belong to the social groups described in the section on Categorization of Social Groups.
